# Intravitreal Conbercept Injection with and without Grid Laser Photocoagulation in the Treatment of Diffuse Diabetic Macular Edema in Real-Life Clinical Practice

**DOI:** 10.1155/2016/2143082

**Published:** 2016-09-29

**Authors:** Yule Xu, Ao Rong, Yanlong Bi, Wei Xu

**Affiliations:** Department of Ophthalmology, Tongji Hospital, Tongji University School of Medicine, Shanghai, China

## Abstract

*Purpose*. To evaluate the efficacy of intravitreal conbercept (IVC) plus modified grid laser photocoagulation (MGP) versus IVC alone for treatment of diffuse diabetic macular edema (DDME).* Methods.* In this retrospective study, 51 DDME patients were treated with either IVC alone (IVC group) or IVC plus MGP (combined group) with 12 months of follow-up. The clinical records of those patients were reviewed.* Results.* 26 patients (31 eyes) received IVC alone and 25 patients (30 eyes) received combined therapy. At month 12, the mean best-corrected visual acuity (BCVA) letter score improvement was 9.1 ± 4.5 and 7.5 ± 4.2 in the IVC group and the combined group and the mean central retinal thickness (CRT) reduction was 145.1 ± 69.9 *μ*m and 168.5 ± 53.6 *μ*m, respectively. There was no statistically significant difference of improvement in BCVA (*P* = 0.164) and decrease in CRT (*P* = 0.149) between the two groups. The mean number of injections delivered was significantly higher (*P* < 0.001) in the IVC group (5.6 ± 0.8 per eye) than in the combined group (3.3 ± 1.2 per eye).* Conclusions*. IVC alone or combined with MGP appeared to be effective for treatment of DDME, achieving the similar clinical efficacy. Moreover, MGP helps to reduce the number of injections.

## 1. Introduction

Diabetic retinopathy (DR) is the most frequent and severe ocular complication of diabetes mellitus, the leading cause of blindness in the working age population [1]. Diabetic macular edema (DME) is a common manifestation of diabetic retinopathy that can occur at any stage of the disease and produce loss of central vision in individuals with diabetes [2, 3].

DME consists of two main forms: diffuse and focal [4]. Diffuse diabetic macular edema (DDME) is characterized by widespread vascular leakage from dilated hyperpermeable capillaries and microaneurysms at the posterior pole, with the result of widespread thickening of the macular area. On the other hand, focal diabetic macular edema is typically related to localized area of retinal thickening due to microaneurysms. In DDME, the breakdown of the inner and outer blood-retina barriers is extensive, and its treatment is more challenging than that of focal edema [5, 6].

The Early Treatment Diabetic Retinopathy Study (ETDRS) has demonstrated the value of focal/grid laser treatment in preventing or reversing visual loss from DME [7]. Its beneficial effect is believed to be caused by the induction of proliferation of both the endothelial cells in retinal capillaries and the retinal pigment epithelial cells, thus improving the efficacy of both the inner and outer blood-retina barriers [8]. Several studies have been conducted to use modified grid laser photocoagulation (MGP) for the treatment of DDME [6, 9]. However, DDME is refractory to macular photocoagulation and having a poor visual prognosis. Lee and Olk have demonstrated that visual acuity was stabilized in 60.9%, deteriorated in 24.6%, and improved in only 14.5% of the eyes with DDME after MGP [6]. Furthermore, the treated eyes showed a high rate of recurrence or persistence of macular edema [6, 9].

Vascular endothelial growth factor (VEGF) has been proved to be an angiogenic inducer and a vascular permeability factor, which increases the retinal vascular permeability. Recently, anti-VEGF agents (e.g., bevacizumab, ranibizumab, and aflibercept) have been reported to be effective in reducing DME and improving the best-corrected visual acuity (BCVA) when injected intravitreally [10–15]. Although the use of anti-VEGF drugs is increasingly prevalent, the therapeutic effect seems to be transient and macular swelling tends to recur after a single injection of anti-VEGF drug. Frequent intravitreal injections of anti-VEGF drugs are required to control macular edema. Therefore, it is clinically important to find longer duration anti-VEGF drugs or techniques to reduce the recurrence of DME and to avoid the need for repeated injections.

The most recent anti-VEGF drug is conbercept, also named KH902 (Chengdu Kanghong Biotech Co., Ltd., Sichuan, China), which is a recombinant fusion protein containing the second immunoglobulin (Ig) domain of VEGF receptor 1 (VEGFR1) and the third and the fourth Ig domains of VEGFR2 and the Fc region of human IgG [16]. Its affinity for VEGF is 50 times that of bevacizumab and 30 times that of ranibizumab [16, 17]. Compared with aflibercept, it has a lower VEGF dissociation rate, higher binding affinity, decreased adhesion to extracellular matrix, and a lower isoelectric point that results in a longer clearance time [18]. The half-life of conbercept has not been calculated in human eyes, but in rabbit eyes it is demonstrated to be 4.2 days. It was shown that a single injection of conbercept in rabbits reduced the ocular-free VEGF concentration over 60 days [19]. Intravitreal administration of conbercept has been shown to prevent choroidal neovascularization (CNV) growth and leakage in nonhuman primate [16, 17]. Several studies and clinical trials have demonstrated the safety and efficacy of conbercept in treating patients with neovascular age-related macular degeneration (AMD) [20–22]. Recently, the China Food and Drug Administration has approved conbercept for the treatment of neovascular AMD. In addition, conbercept suppresses high glucose-induced migration and sprouting of human retinal endothelial cells (HRECs) through not only binding VEGF, but also placental growth factor (PlGF) to inhibit the activation of Src-Akt1-Erk1/2 pathway [23]. Conbercept is a drug that potentially inhibits angiogenic pathways involved in DR and could be beneficial for DME. Solaiman et al. have reported that a combined therapy with intravitreal bevacizumab (IVB) and sequential MGP after 3 weeks appeared to be superior to MGP or IVB alone for the treatment of DDME [24]. Because intravitreal anti-VEGF drugs and MGP achieve their favorable effect through different pathways, a combination of both, theoretically, could be more effective than either treatment alone.

Thus, this study was conducted to evaluate the efficacy of intravitreal conbercept (IVC) plus MGP versus IVC alone in the treatment of DDME in real-life clinical practice.

## 2. Materials and Methods

This was a retrospective study performed at the Department of Ophthalmology of the Tongji Hospital affiliated to Tongji University (Shanghai, China). We reviewed the clinical records of 51 consecutive patients (61 eyes) with DDME treated with IVC alone or IVC plus MGP. All the patients received initial injection from 01.04.2014 to 31.03.2015 and had 12 months of follow-up. This study was approved by the Review Board/Ethics Committee of the Tongji Hospital and was conducted according to the ethical standards laid down in the 1964 Declaration of Helsinki. After discussion with the patient regarding the benefits, risks, and alternatives to treatment, informed consent was obtained from each participant.

In this study, DDME was defined as having two or more disk areas of retinal thickening and involving part of the foveal avascular zone [25]. The diagnosis of DDME was based on the characteristic clinical, fluorescein angiographic (FA), and optical coherence tomography (OCT) features. All patients with DDME treated with IVC alone (IVC group) or IVC plus MGP (combined group) were included in this study. The pattern of treatment was determined at the discretion of the treating physician. Participants were aged ≥18 years and diagnosed with either type 1 or type 2 diabetes mellitus. The glycosylated hemoglobin (HbA1c) was controlled at ≤10% for at least 3 months before involvement in the study and during the treatment period. Visual impairments were due to DDME and no other causes in the opinion of the investigator. All eyes included in the study should have a central retinal thickness ≥300 *μ*m measured by OCT and best-corrected visual acuity (BCVA) letter score between 78 and 24 measured by the ETDRS protocol. Exclusion criteria included previous macular laser photocoagulation, macular traction as evidenced by fundus biomicroscopy and OCT, macular ischemia as diagnosed by FA, prior intraocular injection of anti-VEGF or steroids, history of glaucoma or ocular hypertension, presence of proliferative diabetic retinopathy (PDR) with high-risk characteristics, significant media opacity, and previous panretinal laser photocoagulation or intraocular surgery performed within the last 6 months. Patients with uncontrolled hypertension and recent thromboembolic events were also excluded.

Patients in both groups were started on the intravitreal injection of 0.5 mg conbercept. The retreatment of IVC was performed whenever there was a recurrence of DDME. Recurrence was defined as a decrease of BCVA associated with OCT evidence of increasing macular thickness (≥50 *μ*m) and/or an increase of diffuse fluorescein leakage involving the center of the macula on FA. In the combined group, MGP was performed 3 weeks after the initial IVC injection. Supplemental laser treatments were performed at a minimum of 3 months from the previous laser treatment if OCT and/or FA confirmed the presence of residual or recurrent DDME.

All patients were followed up for at least 12 months, and study visits were scheduled monthly. Before each treatment and during the follow-up period, they received a complete ophthalmic examination including BCVA, slit-lamp biomicroscopy, dilated funduscopic examination, fundus photography, and OCT. FA was performed at baseline and before repeated IVC or at the discretion of the examiner. BCVA was assessed using the ETDRS visual acuity chart at 4 meters. The central retinal thickness (CRT) was measured by Cirrus HD-OCT (Carl Zeiss, Meditec, Dublin, CA) utilizing six diagonal slow 6 mm radial line scans, with software version 4.0. The CRT of the 1 mm central retina was obtained using the macular thickness map for our calculations.

Intravitreal conbercept injections were performed in the operating room under the complete aseptic conditions with topical anesthesia and insertion of a lid speculum. After disinfection, 0.05 mL of solution containing 0.5 mg of conbercept was injected intravitreally 3.5 to 4 mm posterior to the limbus with a 30-gauge needle through the superotemporal quadrant. After the treatment, patients were examined at day 1 and day 7 and each month thereafter. All eyes underwent an ophthalmic examination for anterior chamber (AC) reaction and intraocular pressure (IOP) rise. All ocular and systemic adverse events (AEs), including information on their relationship to study drug and procedure, were recorded at the visits.

Modified grid laser photocoagulation (MGP) was performed by one trained ophthalmologist with double-frequency Nd:YAG (532 nm) laser. After topical anesthesia and placement of a contact lens, two to three rows of 100 *μ*m spots were applied to the parafoveal region, where there was retinal thickening up to and including the edge of the foveal avascular zone; the lesions were placed 100 *μ*m apart. Then, 150 *μ*m to 200 *μ*m spots were placed approximately 200 *μ*m apart and were applied to all remaining areas of retinal thickening and in all areas of capillary nonperfusion [26]. Laser treatment usually induced a light grayish color change in retina.

Primary outcome measures included the changes in BCVA and CRT. Secondary outcome measures were the mean number of injections needed during the 12-month study period.

Statistical analysis was performed using SPSS software version 17.0 (SPSS, Inc., Chicago, IL, USA). Qualitative variables were stated using percentage, and quantitative data were reported as mean ± standard deviation (SD). Differences in categorical variables were assessed with the chi-square test. The paired samples* t*-test was performed to compare the BCVA and the CRT to baseline values within each treatment group. The independent samples* t*-test was used to determine statistically significant differences between two groups as regards mean change in BCVA and CRT, as well as mean number of injections consumed. All statistical tests were 2-sided. A *P* value less than 0.05 was considered to be statistically significant.

## 3. Results

We reviewed the clinical records of 51 consecutive patients (61 eyes) with DDME. All patients had 12 months of follow-up. 31 eyes of 26 patients were treated with IVC alone, and 30 eyes of 25 patients were treated with IVC plus MGP. The mean age of the participants was 61.4 ± 11.7 years; 49.0% were women, and 51.0% were men. A total of 94.1% of the participants had type 2 diabetes, and the mean duration of diabetes was 13.6 ± 4.7 years. The mean visual acuity letter score at baseline was 47.4 ± 10.2, and the mean CRT was 483.4 ± 95.7 *μ*m. The baseline ocular and systemic characteristics of each treatment group were compared and presented in Table 1.

**Figure 1:**
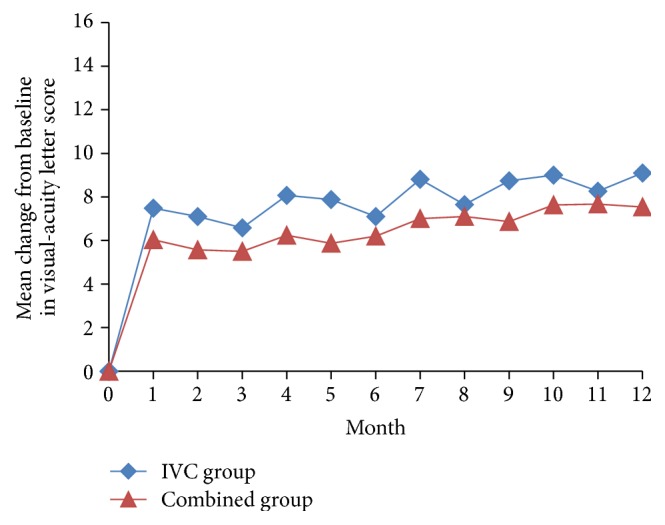
Mean change in best-corrected visual acuity (BCVA) over 12 months. IVC: intravitreal conbercept.

One month after treatment, statistically significant (*P* < 0.001) improvements in the BCVA letter score were observed for both the IVC group (7.5 ± 5.1) and the combined group (6.0 ± 3.9). These improvements were maintained throughout the 12 months of follow-up (Figures 1 and 3). At month 12, the improvement in the BCVA letter score was 9.1 ± 4.5 in the IVC group (*P* < 0.001) and 7.5 ± 4.2 in the combined group (*P* < 0.001). There was no statistically significant difference of improvement in BCVA between both groups (*P* = 0.164, Table 2). Furthermore, at month 12 follow-up visit, 25 eyes (80.6%) gained ≥5 ETDRS letters, 14 eyes (45.2%) gained ≥10 ETDRS letters, and 4 eyes (12.9%) gained ≥15 ETDRS letters in the IVC group; and 22 eyes (73.3%) gained ≥5 ETDRS letters, 10 eyes (33.3%) gained ≥10 ETDRS letters, and 2 eyes (6.7%) gained ≥15 ETDRS letters in the combined group (Table 2).

**Figure 2:**
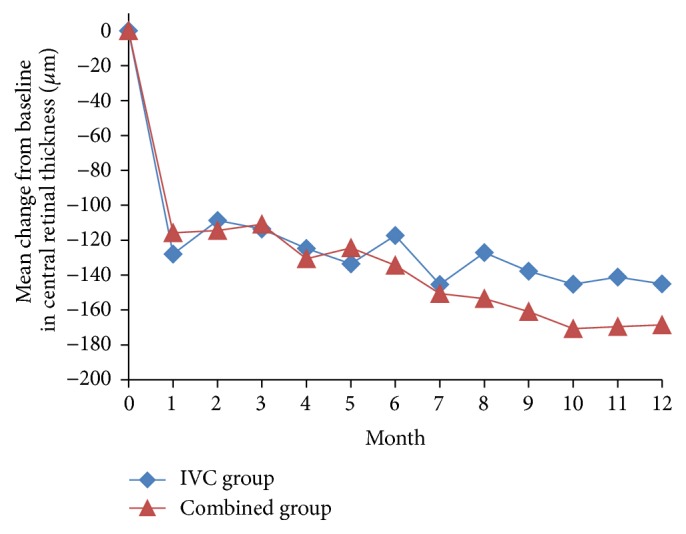
Mean change in central retinal thickness (CRT) over 12 months. IVC: intravitreal conbercept.

**Figure 3:**
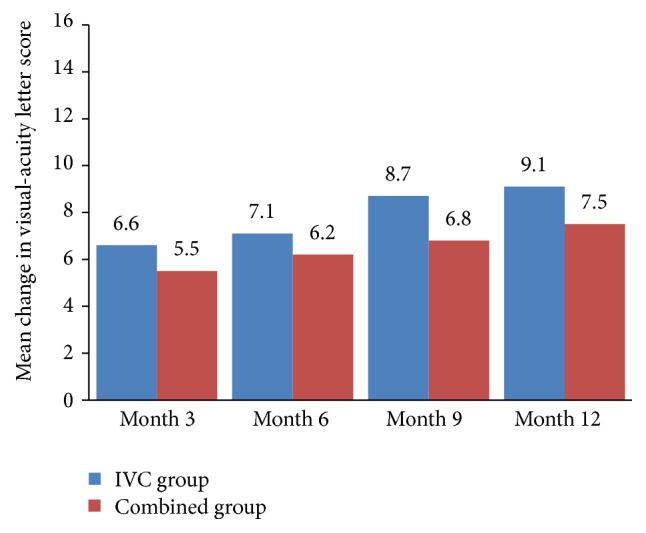
Mean change in best-corrected visual acuity (BCVA) at months 3, 6, 9, and 12. IVC: intravitreal conbercept.

**Figure 4:**
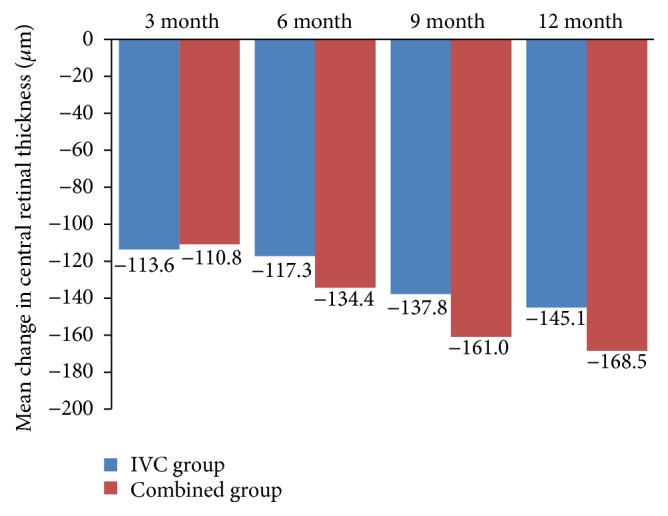
Mean change in central retinal thickness (CRT) at months 3, 6, 9, and 12. IVC: intravitreal conbercept.

We also found a decrease in CRT in two groups from baseline to the 12 months of follow-up (Figures 2 and 4). At month 12, the mean CRT reduction was 145.1 ± 69.9 *μ*m in the IVC group and 168.5 ± 53.6 *μ*m in the combined group. There was no statistically significant difference of decrease in CRT between the two groups (*P* = 0.149, Table 2).

In our study, the mean number of injections consumed was significantly higher (*P* < 0.001) in the IVC group (5.6 ± 0.8 per eye) than in the combined group (3.3 ± 1.2 per eye) throughout the study period. The numbers of the macular MGP procedures performed were 2.1 ± 0.6 in the combined group. In the IVC group, 3 eyes (9.7%) received 7 injections, 14 eyes (45.2%) received 6 injections, 12 eyes (38.7%) received 5 injections, and 2 eyes (6.4%) received only 4 injections over the 12 months of study period. In the combined group, 2 eyes (6.7%) received 6 injections, 3 eyes (10.0%) received 5 injections, 5 eyes (16.6%) received 4 injections, 15 eyes (50.0%) received 3 injections, 3 eyes (10.0%) received 2 injections, and 2 eyes (6.7%) received 1 injection.

There were no reports of serious complications related to the intravitreal injection during the 12 months of follow-up, such as rhegmatogenous detachment or endophthalmitis. Subconjunctival hemorrhage was reported in 9 eyes (29.0%) in the IVC group and in 7 eyes (23.3%) in the combined group. Mild vitreous hemorrhage was reported in 1 eye (3.2%) in the IVC group. No systemic adverse events were observed in both groups throughout the study duration.

## 4. Discussion

This is a single-center retrospective study that evaluates the anatomical and functional outcomes during 12 months of follow-up in patients with DDME treated with either IVC or IVC plus MGP. Our results indicated that intravitreal injection of 0.5 mg conbercept with and without MGP provided fast decrease in CRT and improvement in BCVA at month 1, and these changes were maintained throughout 12 months of follow-up. At the end of the 12 months, there was no statistically significant difference of improvement in BCVA (*P* = 0.164) and decrease in CRT (*P* = 0.149) between two groups.

This study results are consistent with the results of the similar studies. Lee et al. reported no significant differences between a bevacizumab injection only treatment group and a bevacizumab injection plus macular laser photocoagulation (MPC) combination treatment group with 6 months of follow-up. Bevacizumab plus MPC combination treatment could maintain visual acuity and reduce the recurrence of macular edema [27]. In the RESTORE study [28], three monthly injections of ranibizumab of 0.5 mg and then as needed either alone or combined with laser therapy were more effective than laser alone in improving functional and anatomical outcomes in patients with visual impairment associated with DME. However, no efficacy differences were detected between the ranibizumab alone and ranibizumab plus laser arms of this trial. In the DRCR.net-1 trial [10, 29], four monthly injections of ranibizumab of 0.5 mg and then as needed combined with prompt or deferred laser were more effective than prompt laser alone in improving both functional and anatomical outcomes in patients with visual impairment associated with DME. The efficacy of ranibizumab plus prompt laser appeared to be similar to that of ranibizumab plus deferred laser.

Previous studies have demonstrated the efficacy of intravitreal injection of anti-VEGF drug in patients with DME [10–15]. However, these beneficial effects were transient, and recurrence of macular edema was observed within a few weeks after the treatment when anti-VEGF drug disappeared from the vitreous. In our study, the average number of intravitreal injections was 5.6 ± 0.8 in the IVC group during 12 months of follow-up, which indicated that the mean duration of the treatment-free interval was approximately 2.1 months.

In our study, we also found that the mean number of injections was significantly lower (*P* < 0.001) in the combined group (3.3 ± 1.2 per eye) than in the IVC group (5.6 ± 0.8 per eye) throughout the study period, which meant that combining IVC with MGP could decrease the rate of recurrence of DDME, the frequency of repeated IVC injections, and the possible adverse effects associated with intravitreal injections. This matches with the results of the study conducted by Solaiman et al. [30]. They provided repeated intravitreal injections of bevacizumab with and without macular grid photocoagulation for the treatment of 22 patients with bilateral DDME. By the end of the follow-up duration (14.2 ± 1.91 months), the mean number of injections was significantly lower (*P* < 0.05) in the combined group (2.36 per eye) than in the IVB group (3.27 per eye).

The pathophysiology of DME is complex and multifactorial. Chronic hyperglycemia, protein kinase C formation, free radical accumulation, advanced glycation end-product proteins, and ischemia-driven release of vascular endothelial growth factor (VEGF) are some factors that contribute to chronic capillary damage and increased permeability [31]. Anti-VEGF drug expressly aims at halting the effects of VEGF on retinal and vascular structures, but it does not alleviate macular hypoxia. Laser treatment can destroy some photoreceptors and induce a complex action on the retinal pigment epithelium, thus leading to reduction of oxygen consumption, increase in the inner retinal oxygen levels, and improvement of retinal hypoxia [32]. Hence, when MGP is used in conjunction with IVC, it helps in reducing the macular hypoxia and decreasing the rate of recurrence of DME and prolonging the effect of IVC. Furthermore reduction in macular thickness and restoration of retinal transparency achieved by IVC facilitate laser treatment and reduce the need for high laser energy [33]. Thus, each treatment modality is potentiating the effect of the other because of the different modes of action. Intravitreal treatment might rapidly reduce macular edema and lead to more rapid visual acuity improvement, whereas slower benefit accrues over time as a result of laser treatment.

Limitations of our study include that it is nonrandomized, retrospective, and single-centered, which precludes any estimation of the efficacy or safety of IVC with or without MGP. A larger size sample and a longer follow-up period would be ideal to verify and confirm the results of the present study, to select the correct timing for MGP after IVC, and also to establish parameters for selection of patients who are most suitable for undergoing combined therapy. Moreover, there was no standardized adverse event form to collect the safety data in our study. Despite these limitations, our results are very promising and suggest the possible needs for the further investigations.

In conclusion, this preliminary study showed that IVC injections both with and without MGP are effective in the treatment of DDME, achieving the similar clinical efficacy. The additional MGP reduces the number of the repeated intravitreal injections, which not only decreases the possible adverse effects of the intravitreal injections, but also minimizes the financial cost. Evaluation in a multicenter randomized controlled clinical trial with longer follow-up is needed to evaluate the safety and efficacy of IVC plus MGP versus IVC alone for treatment of DDME.

## Figures and Tables

**Table 1:** Baseline characteristics of patients with DDME included in two treatment groups.

Characteristic	IVC group	Combined group	*P* value
Number of patients, *n*	26	25	—
Number of eyes, *n*	31	30	—
Mean age ± SD (years)	60.9 ± 12.9	62.0 ± 10.5	0.746^*∗*^
Gender, *n* (%)			
Men	12 (46.2)	14 (56.0)	
Women	14 (53.8)	11 (44.0)	0.482^*∗∗*^
Diabetes type, *n* (%)			
Type I	2 (7.7)	1 (4.0)	
Type II	24 (92.3)	24 (96.0)	—
Mean HbA1c ± SD	8.0 ± 0.9	7.9 ± 0.8	0.560^*∗*^
Mean duration of diabetes ± SD (years)	14.0 ± 5.5	13.1 ± 3.7	0.526^*∗*^
Mean duration of DDME ± SD (months)	4.9 ± 3.8	4.7 ± 2.8	0.812^*∗*^
Mean BCVA ± SD (letter score)	48.8 ± 10.0	45.9 ± 10.4	0.283^*∗*^
Mean CRT ± SD (*μ*m)	487.2 ± 101.0	479.3 ± 91.4	0.750^*∗*^

DDME: diffuse diabetic macular edema; IVC: intravitreal conbercept; SD: standard deviation; HbA1c: hemoglobin A1c; BCVA: best-corrected visual acuity; CRT: central retinal thickness.

^*∗*^Independent *t*-test; ^*∗∗*^chi-square analysis.

**Table 2:** Best-corrected visual acuity and central retinal thickness outcome at month 12.

Characteristic	IVC group (*n* = 31)	Combined group (*n* = 30)
Mean BCVA letter score at month 12 ± SD	57.9 ± 9.4	53.5 ± 11.8
Mean CRT at month 12 ± SD, *μ*m	342.1 ± 76.9	310.8 ± 81.0
Mean change in BCVA letter score from baseline to month 12		
Mean ± SD	9.1 ± 4.5	7.5 ± 4.2
*P* value	0.164^*∗*^	
Mean CRT change from baseline to month 12 ± SD, *μ*m		
Mean ± SD	−145.1 ± 69.9	−168.5 ± 53.6
*P* value	0.149^*∗*^	
Categorized BCVA letter score outcome at month 12, *n* (%)		
Gain of ≥5	25 (80.6)	22 (73.3)
Gain of ≥10	14 (45.2)	10 (33.3)
Gain of ≥15	4 (12.9)	2 (6.7)

IVC: intravitreal conbercept; BCVA: best-corrected visual acuity; CRT: central retinal thickness; SD: standard deviation.

^*∗*^Independent *t*-test.
